# Single and combined use of *Cannabis sativa* L. and carbon-rich materials for the removal of pesticides and endocrine-disrupting chemicals from water and soil

**DOI:** 10.1007/s11356-020-10690-7

**Published:** 2020-09-14

**Authors:** Elisabetta Loffredo, Giuseppe Picca, Marco Parlavecchia

**Affiliations:** Dipartimento di Scienze del Suolo, della Pianta e degli Alimenti, Università degli Studi di Bari Aldo Moro, Via Amendola 165/A, 70126 Bari, Italy

**Keywords:** Hemp, Phytoremediation, Biochar, Compost, Endocrine disruptor, Water Decontamination, Plant uptake, Organic contaminant

## Abstract

Hemp (*Cannabis sativa* L.) seedlings were used to remove from water the fungicide metalaxyl-M and the endocrine disruptor (EDC) bisphenol A (BPA) at concentrations ranging from 2 to 100 μg mL^−1^. In 7 days of exposure, despite the phytotoxicity of each compound that reduced elongation and biomass, the seedlings were able to remove between 67 and 94% of metalaxyl-M and between 86 and 95% of BPA. The amounts of metalaxyl-M and BPA extracted from plant dry biomass were in the range of 106–3861 μg g^−1^ and 16–101 μg g^−1^, respectively, and resulted positively correlated to both the dose of compound added (*P* ≤ 0.01) and the amount removed by the plants (*P* ≤ 0.01). Plant uptake and transformation were the main mechanisms involved in the removal of the compounds. In another set of experiments, hemp was used to remove a mixture of two pesticides, metalaxyl-M and metribuzin, and three EDCs, BPA, 17β-estradiol (E2), and 4-tert-octylphenol (OP), at concentrations of 10, 10, 10, 10, and 1 μg g^−1^, respectively, from soil column not added and added with 2.5% (w/w) of a green compost (CM) or a wood biochar (BC). In 25 days, plants did not alter considerably the distribution of the compounds along the soil profile and were capable of removing, on average, 12, 11, 10, 9, and 14% of metalaxyl-M, metribuzin, BPA, E2, and OP, respectively. During growth, hemp transformed the compounds and accumulated part of them (except OP) mainly in the shoots. CM and, especially, BC significantly protected the plants from the toxicity of the compounds and enhanced the retention of the latter in soil, contrasting leaching. Thus, the single or synergistic use of hemp and amendments deserves attention being a very low-cost and eco-sustainable strategy to remediate water and soil.

## Introduction

A primary concern of the last years is the increasing presence in the environment of the so-called emerging pollutants (EPs). EPs are synthetic or naturally occurring chemicals that are not commonly monitored in the environment, but that have the potential to enter into it causing known or suspected adverse ecological and human health effects (Geissena et al. 2015). Pesticides, pharmaceuticals, wood preservatives, industrial product and by-products, and dyes are examples of EPs.

A major risk related to these compounds is their high leaching potential, especially for those with low hydrophobicity like some pesticides, thus contaminating groundwater (Arias-Estévez et al. 2008). Metalaxyl-M (methyl *N*-(2,6-dimethylphenyl)-*N*-(methoxyacetyl)-d-alaninate)) is the biologically active R-enantiomer of the racemic compound metalaxyl. It is one of the most used fungicide worldwide and is highly persistent, mobile, and leachy in soil (Fernandes et al. 2003). Metribuzin (4-amino-6-tert-butyl-3-methylsulfanyl-1,2,4-triazin-5-one) is a selective triazinone herbicide. Because of the high water solubility, it has been included by the United States Environmental Protection Agency into the group of pesticides that have the greatest potential for leaching into groundwater (USEPA 2003).

Among EPs, the endocrine-disrupting chemicals (EDCs) have drawn increasingly extensive attention. These compounds are currently ubiquitous in the environment, food, and consumer products and can have severe impact on the endocrine functions of animals, especially aquatic, and humans (Diamanti-Kandarakis et al. 2009). EDCs may enter surface water, groundwater, and soil through agricultural practices and the application, discharge, and disposal of urban and industrial effluents, sludges, and other wastes. The environmental fate of some endocrine disruptors has been extensively investigated (Baronti et al. 2000; Ying et al. 2002; Campbell et al. 2006).

The xenoestrogen bisphenol A (2,2-(4,4 dihydroxydiphenyl) propane, BPA) is one of the chemicals produced in the highest quantities worldwide, with almost 3 million tons produced each year (Vandenberg et al. 2009). BPA is the building block of epoxy resins and polycarbonates and is adopted as stabilizer for polyvinyl chloride. The 17β-estradiol (17β-estra-1,3,5(10)-triene-3,17-diol, E2) is the major endogenous estrogen in humans, and it is present in several hormonal therapy products. The 4-tert-octylphenol (OP) originates by microbial breakdown from the surfactants octylphenol polyethoxylates. This molecule possesses estrogenic activity and is highly recalcitrant to biodegradation (Ying et al. 2002).

The coexistence in cultivated soil of different classes of contaminants, such as pesticides and EDCs, is very likely to occur due to the current agricultural practices that makes extensive use of waste biomass and wastewaters which are rarely thoroughly decontaminated and plant-protective chemicals.

The necessity to provide sustainable solutions to water and soil pollution has led to develop alternative, cost-effective, and eco-friendly technologies for remediating water and soil from contaminants (Morillo and Villaverde 2017; Wu and Wu 2019). Among the numerous remediation methodologies explored and tested in recent years, bio-based technologies seem to be an interesting economical alternative (Gerhardt et al. 2009). Phytoremediation is a process that exploits plant physiological processes, such as transpiration, root exudation, absorption, translocation, and metabolization, and rhizosphere microorganisms to remove mineral and organic pollutants from water, soil, sludge and sediments, both in situ and ex situ. There are various phytoremediation strategies whose choice depends on the type of pollutants and their concentrations, the matrix, and the plant species adopted (McCutcheon and Schnoor 2003; Gerhardt et al. 2009). Phytoextraction or phytoaccumulation consists in absorbing pollutants by the root system and eventually translocate them to the aerial organs, ensuring the permanent decontamination of the site. This technique has been extensively applied to remove metals but also pesticides (Gasco et al. 2019; Tarla et al. 2020). Rhizofiltration is typical not only of aquatic plant species which uptake pollutants from aquatic environments but also of terrestrial plants, such as sunflower or hemp, that have an extended root system and large root surface area (Gerhardt et al. 2009). Phytostabilization or phytoimmobilization relies on the ability of plants to decrease the mobility or bioavailability of pollutants, avoiding their leaching and possible entrance into groundwater or food chain. However, the latter mechanism that concern especially heavy metals does not allow the elimination of pollutants from the contaminated matrix. A relevant role for organic pollutants has played by phytodegradation or phytotransformation. This process regards the capacity of some plants to degrade or transform partially or completely organic contaminants by means of their enzymes. Rhizodegradation concerns an indirect action of plants which produce root exudates containing an easily degradable C-source that stimulate the degradative metabolism of microorganisms in the rhizosphere, acting also on recalcitrant pollutants.

An added value of phytoremediation is that plants give a relevant contribution to soil fertility both directly, through root expansion and exudation, and indirectly promoting the growth and activity of microorganisms (Paz-Ferreiro et al. 2014).

Hemp (*Cannabis sativa* L.) is an annual herb used in many types of non-food industries. Its high biomass, deep-root and short life cycle, and ease of adaptation to different climatic conditions make it very suitable for phytoremediation. Moreover, hemp has a very high capacity to absorb and accumulate organic and inorganic pollutants (Linger et al. 2002).

Despite its great potential, phytoremediation could have some application limits, especially in heavily contaminated areas, due to the phytotoxicity of pollutants, often present in combination, that could seriously compromise plants survival and growth (McCutcheon and Schnoor 2003). Trying to overcome this problem, increasing attention has been given to the so-called assisted or enhanced phytoremediation that exploits the synergistic combination of plants and C-rich materials (Paz-Ferreiro et al. 2014; Lu et al. 2015; Chirakkara and Reddy 2015; Gasco et al. 2019). This combined strategy has been applied to depollute soil from heavy metals (Paz-Ferreiro et al. 2014; Lu et al. 2015; Gasco et al. 2019) or mixtures of organic and inorganic contaminants (Chirakkara and Reddy 2015).

The addition to the soil of C-rich materials during the phytoremediation could slow down the decontamination process because of the high sorption efficiency of these materials that may attenuate the availability of contaminants to soil-resident degrading microorganisms. However, there are some aspects that make this double approach worthy to be explored. One aspect concerns the plant-protective activity of these materials, including a pathogen-suppressive activity, that sustains plant mass production and is very important when plants have to face stressful situations in heavily polluted soils, especially in their early growth stage (Gasco et al. 2019). The precarious conditions of remediating plants make them much more fragile, compromising plant survival and consequently the success of phytoremediation. Furthermore, plant candidates for phytoremediation, like hemp, have excellent capacity to uptake pollutants with the water flow and are supposed to be able to effectively compete with the amendments for the retention of contaminants (McCutcheon and Schnoor 2003; Gasco et al. 2019).

The importance of these aspects is the rationale for choosing C-rich materials, including compost (CM) and biochar (BC) to sustain plant remediation. Green CM produced from the wastes of the maintenance of public and private greenery, residues of crops or wood processing, has a remarkable ability to reduce the mobility of organic contaminants in soil (Marín-Benito et al. 2018). BC is the stable carbonaceous by-product obtained from thermochemical conversion, at temperatures ranging from 200 to 800 °C, of biomass in limited oxygen conditions (pyrolysis) to produce bio-oil and syngas (IBI 2015). BC, originally designed for carbon sequestration and soil amendment, has proven to be a very efficient low-cost adsorbent of organic and inorganic contaminants and has been increasingly used for soil and water remediation (Zhang et al. 2013; Loffredo and Taskin 2017; Gasco et al. 2019; Parlavecchia et al. 2019; Zheng et al. 2020).

The objectives of this work were to evaluate (i) the potential of hemp to remove metalaxyl-M and BPA from water during germination and early growth and (ii) the effectiveness of hemp, a green CM and a wood BC, individually or in combination, to remediate a soil multi-contaminated with two pesticides and three EDCs and to influence the leaching of these compounds.

## Materials and methods

### Chemicals, plant, soil, and materials

Metalaxyl-M and metribuzin, both at a purity ≥ 98.0%, bisphenol A (BPA) at 99% purity, 17β-estradiol (E2) at 99.9% purity, and 4-tert-octylphenol (OP) at 99.5% purity were provided by Sigma-Aldrich S.r.l., Milano, Italy. Chemical properties of the compounds are shown in Table 1. All other chemicals of extra pure grade were obtained from commercial sources and used without further purification.

**Table 1:**
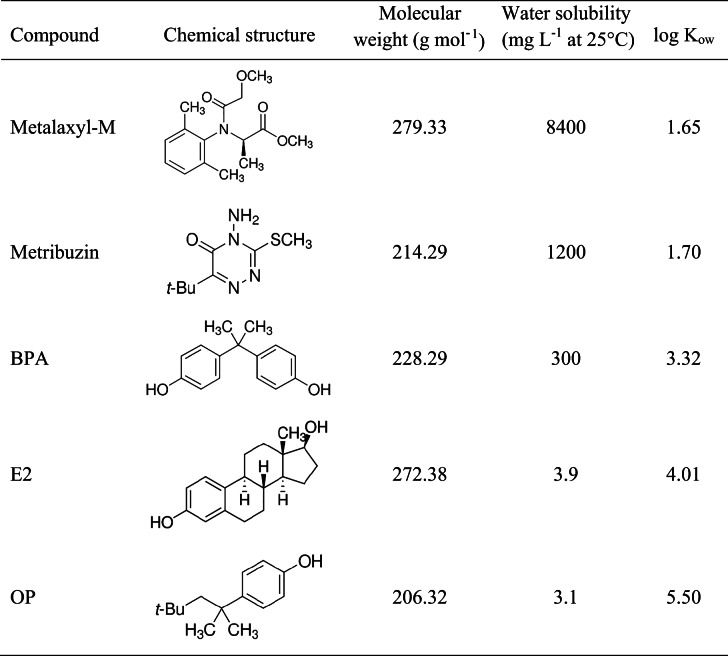
Some properties of the compounds

Data from PubChem open chemistry database at the National Institutes of Health (NIH) (n.d.)

Hemp (*Cannabis sativa* L.) seeds, cultivar Kompolti, were provided by Emporio Canapuglia S.a.s., Conversano, Italy.

The loamy soil used was sampled at 0–20 cm depth at an experimental station located in Southern Italy (41° 1′ N, 16° 54″ E). The soil was air-dried, thoroughly mixed, and 2.0-mm sieved. Some soil properties were determined according to conventional methods. Moisture was determined by heating the soil at 105 °C overnight. The pH was measured suspending the soil in distilled water (soil/H_2_O, 1:2.5, w/v). Electrical conductivity (EC) was measured by a conductivity meter (soil/H_2_O, 1:2, w/v). Soil organic matter (SOM) was determined by the loss on ignition method (Zhang and Wang 2014). Total calcium carbonate was determined by the gas-volumetric method using a Dietrich–Fruhling calcimeter. Moisture, pH, EC, SOM, and total carbonates of soil were, respectively, 5.0% 7.8, 0.23 dS m^−1^, 51.3 g kg^−1^, and 20.9 g kg^−1^.

The CM sample was obtained from the composting process of wastes from public and private greenery and residues of crops and wood processing. CM was produced by Tecnogarden Service S.r.l., Vimercate, Italy, and provided by the Italian Composting and Biogas Association (CIC). Moisture, pH, EC, organic C, and C/N ratio of CM were, respectively, 24%, 7.8, 1.23 dS m^−1^, 270 g kg^−1^, and 15 (data provided by the producer). The BC sample was obtained from vineyard pruning residues through a process of micro-gasification or slow pyrolysis with a thermal maximum of 550 °C and a duration of the process of 3 h, followed by dry cooling. BC was supplied by Blucomb S.r.l., Udine, Italy. Total organic C, moisture, pH, EC, and ash of BC were, respectively, 755 g kg^−1^, 4.5%, 9.9, 2.23 dS m^−1^, and 9.9% (Taskin et al. 2019).

### Removal of the compounds from water

Sets of 10 seeds of hemp were placed on filter paper in Petri dishes (9-cm diameter) and supplied with 3 mL of water (control) or 3 mL of aqueous solutions of metalaxyl-M or BPA at concentrations of 2, 10, 20, 50, and 100 μg mL^−1^. Samples with contaminant solution only (no seeds) were also prepared to evaluate a possible degradation of the compounds during the experimental time. Seed germination and early growth were achieved in a Phytotron growth chamber at 21 ± 1 °C in the dark for 7 days. Then, seedlings were collected and the germination percentage, root and shoot lengths, and fresh and dry weights were measured. The residual amounts of the chemical in the germination medium was measured using high performance liquid chromatography (HPLC) technique (“HPLC analytical procedure” section).

BPA and metalaxyl-M were extracted from the plants according to the procedure described in Ferrara et al. (2006). Briefly, 0.1 g of dry mass was added with 10 mL of pure methanol and kept under mechanical shaking for 4 h. The suspension was then centrifuged for 10 min at 10,000 *g* and an aliquot of 5 mL of the supernatant solution was evaporated to dryness at a temperature of 40 °C using a rotatory evaporator. The residue was dissolved in a volume of 2 mL of acetonitrile/water mixture (70/30, v/v), filtered through 0.45-μm Millipore™ filters and analyzed by HPLC (“HPLC analytical procedure” section).

All experiments were replicated six times and all data obtained were statistically analyzed by one-way analysis of variance (ANOVA) at the 95%, 99%, and 99.9% confidence levels. The means were statistically compared using the least significant difference (LSD) test.

### Removal of the compounds from soil column

A series of columns (30-cm height and 7-cm diameter) were prepared overlapping plexiglass cylinders. The columns were closed at the base with wire mesh and glass wool. Then, 950 g of air-dried soil was poured in the column filling up to a height of 25 cm. In some columns, 9.5 g of the 0–10 cm soil layer was replaced with 9.5 g of CM or BC (2.5% w/w in the 0–10 cm layer). All soil columns were watered up to 60% of the field capacity. After 2 h, a mixture of 2 mg of metalaxyl-M, 2 mg of metribuzin, 2 mg of BPA, 2 mg of E2, and 0.2 mg of OP was incorporated in the upper soil layer (~ 5 cm), achieving concentrations of 10, 10, 10, 10, and 1 μg g^−1^, respectively. After 2 h, six hemp seeds per column were sowed in half of the columns prepared. The treatments obtained were as follows: soil, soil + CM, soil + BC, soil + plants, soil + CM + plants, soil + BC + plants, and uncontaminated soil + plants (control). Then, a volume of 14 mL of distilled water was added to each column (with and without seeds). All treatments and the control were triplicated. During the 25-day experimental period, a volume of 14 mL of water per day (total volume of 350 mL) was added to each soil column. The experiments were performed in a Phytotron growth chamber at a temperature of 25 °C, relative humidity of 70%, and a photoperiod of 14 h (Fig. 2). All biometric data of plants were statistically analyzed by ANOVA at the 95%, 99%, and 99.9% confidence levels. The means were statistically compared using the LSD test.

At the end of experiments, plants were collected, roots were rinsed with distilled water, and root and shoot lengths as well as fresh and dry weights (at 70 °C for 16 h) of plants were measured. The extraction of the compounds from plants was done according to the procedure of Ferrara et al. (2006), briefly described in the “Removal of the compounds from water” section. Samples were finally analyzed by HPLC (“HPLC analytical procedure” section).

The soil columns were sectioned, and the soil of the various sections (0–5, 5–10, 10–15, and 15–25 cm) was separately mixed. An aliquot of 20 g of soil sampled from each section was added with 50 mL of methanol and mechanically shaken for 16 h. Then, the suspension was filtered and an aliquot of 20 mL was centrifuged at 10,000*g* for 10 min. Supernatants were finally analyzed using HPLC (“HPLC analytical procedure” section). The percentages of recovery from soil of metalaxyl-M, metribuzin, BPA, E2, and OP at concentrations of 10, 10, 10, 10, and 1 μg g^−1^ were, respectively, 93.75 ± 2.70, 92.57 ± 1.77, 92.43 ± 0.80, 89.18 ± 2.67, and 91.82 ± 2.08 (*n* = 4). The residual amount of each compound in the whole soil column was calculated as the sum of the amounts extracted in the various sections. The percentages of the compounds disappeared after 25 days in the soil column were calculated as the difference between the initial amounts and those extracted at the end. These data were statistically analyzed by the two-way ANOVA at the 95% confidence level and the means compared using the LSD test.

### HPLC analytical procedure

The HPLC apparatus consisted of a Spectra System™ pump (Thermo Electron Corporation, San Josè, CA, USA) equipped with a Rheodyne 7125 injection valve fitted with a 20-μL loop and connected to a Supelcosil™ LC-18 column (250 mm × 4.6 mm × 5 μm). Using a mixture of acetonitrile/water at a ratio of 70/30 (v/v) as mobile phase and a flow rate of 1 mL min^−1^, the retention time of metalaxyl-M, metribuzin, BPA, E2, and OP were 4.8, 3.8, 7.0, 6.0, and 15.0 min, respectively.

Metalaxyl-M and metribuzin were quantified using a Spectra System UV6000LP™ diode array detector (Thermo Electron Corporation, San Josè, CA, USA) at wavelengths of 220 and 294 nm, respectively. BPA, E2, and OP were quantified using a Spectra SystemFL3000 (Thermo Electron Corporation, San Josè, CA, USA) fluorescence detector operating at wavelengths of 230-nm excitation and 310-nm emission.

The external standard method was adopted to quantify the compounds. Blank samples were prepared for soil and plants in quadruplicate. The matrix effect was investigated by comparing the standards in the appropriate solvent with matrix-matched standards at 1 μg mL^−1^. Results showed that there were not interfering peaks from the matrix and that the retention times of the compounds did not change. All analytes were eluted as separate symmetric peaks. Very good linearities were obtained for all compounds in the ranges 0.05 to 5 μg mL^−1^ and 2 to 100 μg mL^−1^ with *r* values always higher than 0.999. The limit of detection (LOD), calculated considering a signal to noise ratio (S/N) of 3 compared with the background noise of the blank, for metalaxyl-M, metribuzin, BPA, E2, and OP were 0.01, 0.01, 0.01, 0.003, and 0.003 mg L^−1^, respectively. The limit of quantification (LOQ) were 0.03, 0.04, 0.02, 0.01, and 0.01 mg L^−1^ for metalaxyl-M, metribuzin, BPA, E2, and OP, respectively, with S/N = 10.

## Results and discussion

### Removal of the compounds from water

**Fig. 1:**
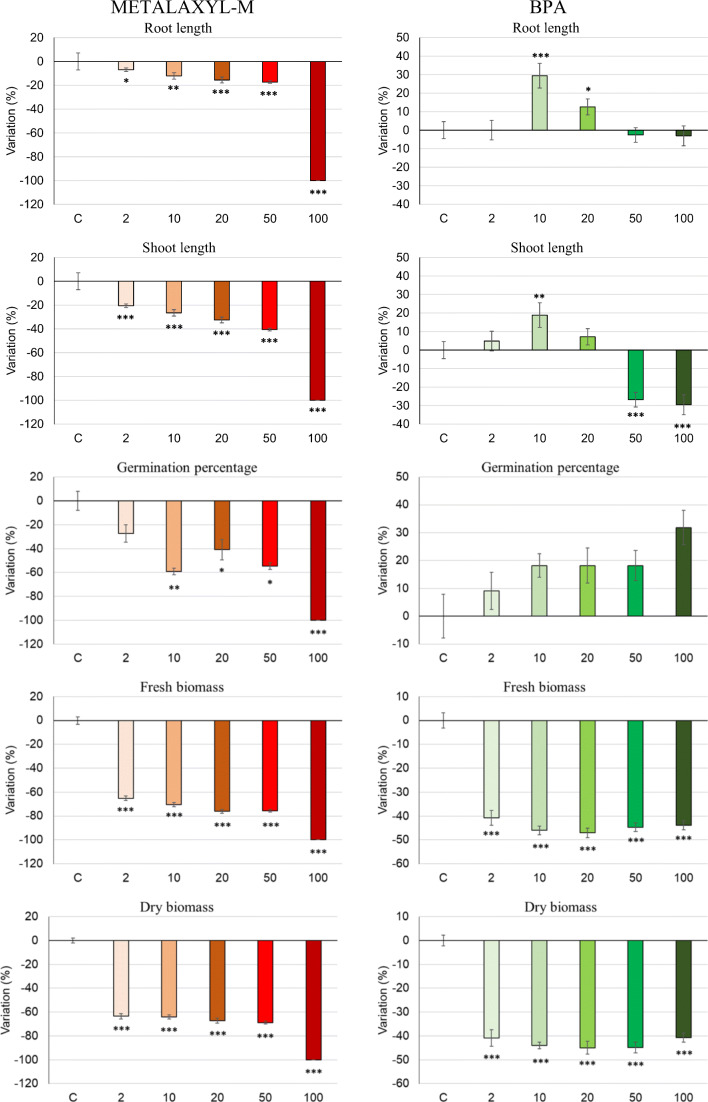
Effects of the dose (μg mL^−1^) of metalaxyl-M and BPA on biometric parameters of 7-day grown hemp seedlings. *P ≤ 0.05, **P ≤ 0.01, ***P ≤ 0.001 according to the least significant difference (LSD) test. The vertical line on each bar indicates the standard error (*n* = 6)

Both BPA and, especially, metalaxyl-M caused some phytotoxic effects on seedlings, reducing shoot and root elongation and fresh and dry biomass (Fig. 1). However, BPA did not affect germination at any concentration and even significantly stimulated root and shoot elongation at a dose of 10 μg mL^−1^ (Fig. 1). On average for the doses applied, metalaxyl-M reduced dry mass of hemp by about 66% (excluding the dose of 100 μg mL^−1^ that completely inhibited germination) and BPA by about 43% (Fig. 1). Teixeira et al. (2011) reported toxic effects on *Solanum nigrum* L. and biomass decrease starting from metalaxyl concentration of 12.5 μg mL^−1^. BPA addition at concentrations of 10 and 50 μg mL^−1^ generally did not inhibit germination and early growth of broad bean, tomato, durum wheat, and lettuce but reduced their dry weights from 10 to 67% at the lower dose and from 17 to 88% at the higher dose (Ferrara et al. 2006).

**Fig. 2:**
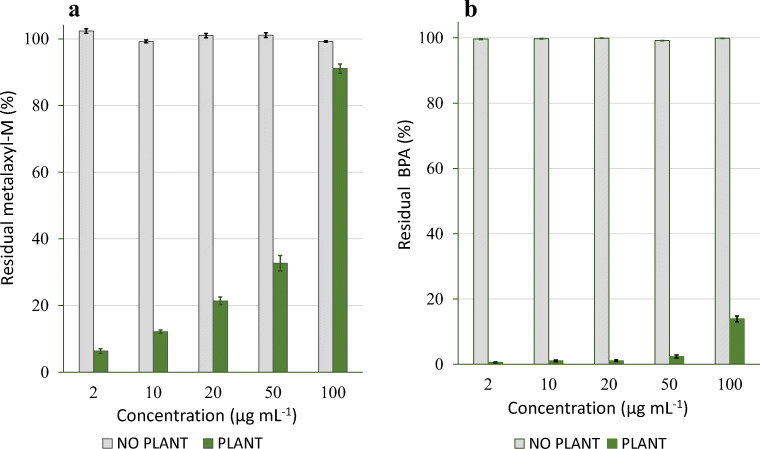
Effects of the dose of metalaxyl-M (**a**) and BPA (**b**) on the residual compound in the medium in the absence and presence of hemp. Data are expressed as percentages of the initial amount of compound added. The vertical line on each bar indicates the standard error (*n* = 6)

Residual amounts of each compound measured in the medium at the end of experiments are shown in Fig. 2. In the absence of plants, at any concentration, no significant decrease of metalaxyl-M and BPA was obtained, denoting that abiotic degradation had a negligible role (Fig. 2). BPA stability in water for several days was reported in previous studies (Imai et al. 2007; Loffredo et al. 2010). Differently, a significant reduction of each compound at each dose was found in the medium with seedlings. Therefore, despite the young plants were stressed by exposure to the chemicals, they were capable of removing considerable amounts of each compound, especially BPA (Fig. 2). Significant positive correlations were calculated between the initial doses of metalaxyl-M (excluding 100 μg mL^−1^) and BPA and the corresponding amounts removed by the seedlings (*P* = 0.006 for metalaxyl-M and *P* = 0.003 for BPA). Residual BPA in the medium with plants was lower than 5% at initial concentrations in the range 2-50 μg mL^−1^ and equal to 14% at the highest concentration (Fig. 2). Imai et al. (2007) found that BPA was rapidly absorbed and metabolized by *Portulaca oleracea*. Seedlings of various herbaceous species, exposed for 5–7 days to 4.6 and 46 μg mL^−1^ BPA concentrations, were capable of removing, respectively, from 25 to 98% and from 30 to 91% of the contaminant (Loffredo et al. 2010). We can suppose that the removal of these contaminants was primarily due to seedling absorption and metabolization and also to a possible plant-promoted microbial degradation in the non-axenic medium. Anyway, this aspect was not investigated in this work. Schmidt and Schuphan (2002), studying BPA metabolism in plant cell culture, demonstrated the rapid uptake of this compound by cells.

**Fig. 3:**
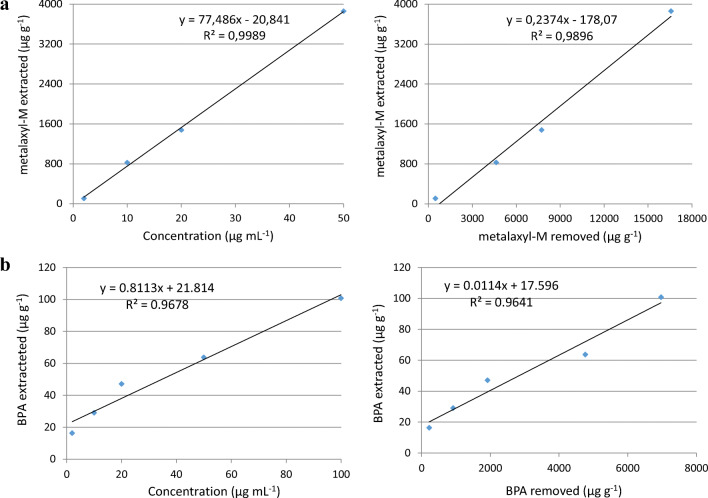
Correlations between the compound concentration added (left) or that removed by plants (right, μg g^−1^ of dry biomass) and the compound accumulated in hemp biomass (μg g^−1^ of dry biomass). **a** Metalaxyl-M. **b** BPA

The amount of compound accumulated in the seedlings exposed for 7 days to various concentrations of metalaxyl-M and BPA, individually, are shown in Table 2. Depending on the initial dose, from 106 to 3861 μg g^−1^ of metalaxyl-M and from 16 to 101 μg g^−1^ of BPA were extracted from dry biomass of seedlings (Table 2). For each compound, the quantity extracted from the seedlings was positively correlated with the initial concentration (*P* ≤ 0.01) and the amount removed by the seedlings (*P* ≤ 0.01) (Fig. 3). Exposing the seedlings to concentrations ranging from 2 to 50 μg mL^−1^, the quantities of metalaxyl-M and BPA accumulated in the tissues were, on average, 20.6 ± 1.3% and 3.6 ± 1.3%, respectively, of the removed amount. Considering similar amounts of compound removed by the seedlings, i.e., 4635 μg g^−1^ of metalaxyl-M and 4766 μg g^−1^ of BPA (Fig. 3), we calculated percentages of accumulation in plants of 17.8 and 1.3%, respectively. Therefore, these results, along with those concerning hemp growth, indicated that, in the same range of concentrations, metalaxyl-M was absorbed much more than BPA, thus producing strong toxicity on seedlings (also lethal) that compromised seriously also its biotransformation. The different response of hemp to the two compounds is reasonably linked to their hydrophobicity, suggesting, in principle, particular attention in the use of hemp for the decontamination of matrices heavily polluted by highly soluble compounds. However, this aspect needs to be explored in adult plants.

**Table 2:** Amount of metalaxyl-M and BPA extracted from hemp seedlings after 7-day growth

Treatment	Product extracted (μg g^−1^ of dry plant mass)
Metalaxyl-M	
2 mg L^−1^	105.56 ± 1.79^a^ a^b^
10 mg L^−1^	825.73 ± 58.25 b
20 mg L^−1^	1477.37 ± 155.72 c
50 mg L^−1^	3860.81 ± 156.28 d
BPA	
2 mg L^−1^	16.28 ± 1.47 a
10 mg L^−1^	29.00 ± 3.23 b
20 mg L^−1^	47.05 ± 6.51 c
50 mg L^−1^	63.66 ± 6.33 d
100 mg L^−1^	100.74 ± 7.48 e

^a^Standard error of the mean (*n* = 6)

^b^Data were statistically treated by the LSD test at *P* ≤ 0.05

Few information is present in the literature on the absorption and accumulation of these contaminants by plants. Gong et al. (2020) ascribed the rapid absorption and translocation of metalaxyl-M in plants to both its hydrophilicity and the small molecular size that allowed an easy passage through cell membrane and Casparian strip. Perennial ryegrass and radish, exposed for 16 days to 4.6 and 46 μg mL^−1^ BPA concentrations, accumulated in dry plant mass, respectively, 0.8 and 2.7% of initial BPA at the lower dose and 0.2 and 0.4 % at the higher dose, indicating an efficient metabolization of this compound (Loffredo et al. 2010).

### Removal of the compounds from soil column

#### Leaching and disappearance in soil

During the experimental period, the five molecules underwent a series of processes in the soil, including absorption by plants, adsorption on the solid fraction, leaching, and degradation. Removal by plants, aside from absorption, was possibly also due to enhanced microbiological degradation stimulated by root exudates (rhizodegradation or phytostimulation), although in this work, the two processes were not investigated individually.

**Fig. 4:**
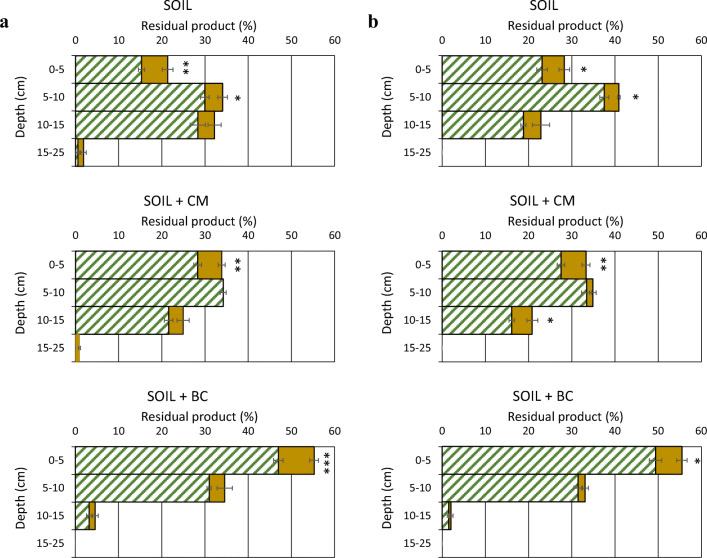
Residual metalaxyl-M (**a**) and metribuzin (**b**) at different depths of soil only and soil added with 2.5% (w/w) of CM or BC. Whole bar: bare soil; striped portion of the bar: soil with plants. Means were separated by the least significant difference (LSD) test. *P ≤ 0.05, **P ≤ 0.01, ***P ≤ 0.001. The horizontal line on each bar indicates the standard error (*n* = 3)

**Fig. 5:**
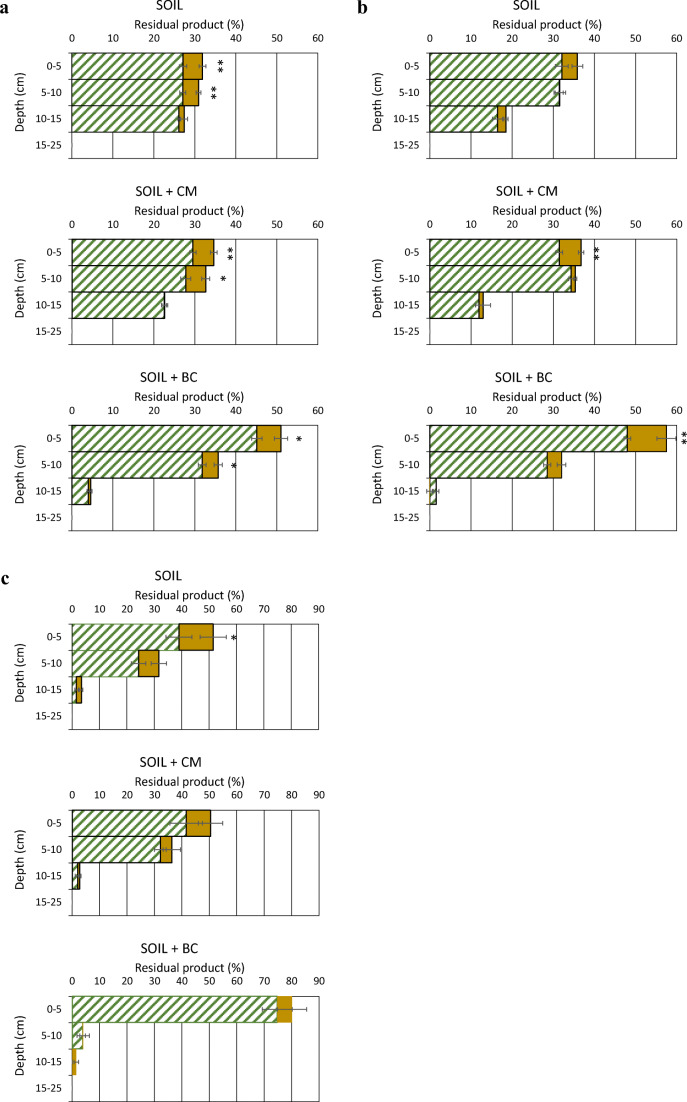
Residual BPA (**a**), E2 (**b**), and OP (**c**) at different depths of soil only and soil added with 2.5% (w/w) of CM or BC. Whole bar: bare soil; striped portion of the bar: soil with plants. Means were separated by the least significant difference (LSD) test. *P ≤ 0.05, **P ≤ 0.01, ***P ≤ 0.001. The horizontal line on each bar indicates the standard error (*n* = 3)

As expected, both materials, especially BC, improved the capacity of soil to retain the molecules in the upper layers, contrasting their leaching. In soil + BC treatments, the maximum amounts of all compounds were found in the 0–5 cm layer (Figs. 4 and 5). Compared with bare soil, the presence of plants did not change substantially the distribution of the chemicals along the soil profile. This could be attributed to the poor extension of the roots both for the short growth time and for the stress of the multi-contamination of soil. Anyway, in various cases, the presence of plants significantly decreased the residual compounds, especially in the upper 0–5 cm layer where most of the roots are present (Figs. 4 and 5).

Among the five compounds, metalaxyl-M was only found in the deepest layer (15–25 cm), albeit in very small quantities (Fig. 4a). The relatively high water solubility and mobility of this molecule can account for its overall leaching. Compared with unamended soil, the addition of CM and, especially, BC altered the distribution of this compound along the soil profile, counteracting its downward movement (Fig. 4a). Fernandes et al. (2003) reported that the adsorption of metalaxyl in soil was affected mainly by the organic fraction. The adsorption capacity of a CM-based biomixture for metalaxyl-M was much higher than that of the soil (Karanasios et al. 2010). Gámiz et al. (2016), studying the effects of an olive-mill waste CM and its BC on metalaxyl movement in soil, concluded that although both materials were able to limit metalaxyl leaching, BC was much more effective. In a recent study, Parlavecchia et al. (2019) compared the adsorption capacity of a non-amended soil and the same soil amended with 2% (w/w) of two types of CM or BC and found that, on average, the retention capacity of the soil for metalaxyl-M was, respectively, twice or four times higher in the amended soil.

Hemp plants were able to significantly reduce residual metalaxyl-M in the upper layers of the soil, compared with bare soil, reducing the risk of leaching (Fig. 4a). That was expected considering that most of the roots were located approximately in the upper 10 cm of soil. When the whole soil column was considered, after 25 days from the start of the experiments, on average, 8% of the amount of metalaxyl disappeared in bare soil, likely due to degradation, and no significant influence of the amendments was observed (Table 3). The presence of hemp reduced noticeably the residual metalaxyl-M in the column, especially in not amended soil where up to 26% of the compound disappeared (Table 3). In planted soil, the removal of this compound was significantly (*P* ≤ 0.05) reduced by both amendments (Table 3). We can hypothesize that the retention of metalaxyl-M by CM and, especially, BC hindered the uptake of this compound by the plants.

**Table 3:** Effects of treatment, plants, and their interaction on the amount of compound disappeared in 25 days in the whole soil column. Data are expressed as percentage of the initial amount added per column

Treatment	Bare soil	Soil + plants	Average
Metalaxyl-M; 0.05P: 5.9^a^			
Soil	11.2 ± 1.3	26.0 ± 2.4	18.6 a
Soil + CM	7.2 ± 1.3	16.0 ± 2.2	11.6 b
Soil + BC	5.6 ± 2.2	18.9 ± 1.1	12.3 b
Average	8.0 b	20.3 a	
Metribuzin^b^			
Soil	7.9 ± 0.7	20.5 ± 0.8	14.2
Soil + CM	10.9 ± 1.4	22.9 ± 1.5	16.9
Soil + BC	9.4 ± 2.0	17.6 ± 0.9	13.5
Average	9.4 b	20.3 a	
BPA^b^			
Soil	10.0 ± 2.0	19.1 ± 1.9	14.5
Soil + CM	10.2 ± 0.9	20.3 ± 1.4	15.3
Soil + BC	8.8 ± 2.3	19.2 ± 1.4	14.0
Average	9.6 b	19.5 a	
E2^b^			
Soil	14.0 ± 2.6	20.0 ± 1.4	17.0
Soil + CM	14.9 ± 2.4	22.3 ± 1.2	18.6
Soil + BC	9.5 ± 4.0	21.9 ± 1.1	15.7
Average	12.8 b	21.4 a	
OP; 0.05P: 7.5^a^			
Soil	13.7 ± 2.5	35.2 ± 5.3	24.4 a
Soil + CM	13.7 ± 4.4	24.4 ± 8.2	19.0 b
Soil + BC	14.4 ± 2.3	21.5 ± 6.1	18.0 b
Average	13.9 b	27.0 a	

Significant differences between means are shown by different letters according to the least significant differences (LSD) test at *P* ≤ 0.05

^a^Least significant difference for the interaction of treatment × plants at *P* ≤ 0.05

^b^Letters or LSD value are not reported for not significant factors or interactions

The leaching of metribuzin down to 15 cm can be attributed to its relatively low hydrophobicity. The presence of CM and, especially, BC favored the retention of this molecule in the upper layer (Fig. 4b). In soil + BC treatment, almost all residual metribuzin was found in the upper 10 cm of soil, especially in the top layer (0–5 cm), where its concentration was 5.84 μg g^−1^ (Fig. 4b). Karanasios et al. (2010) reported the relevant contribution of CM to retain metribuzin, resulting in the Freundlich sorption constant (Kf_ads_) of a compost-containing biomixture up to three times higher than that of the soil. In soil column tests, Lopez-Pineiro et al. (2013) reported that CM amendment of soil noticeably increased the capacity of soil to retain metribuzin, reducing its leaching and also favoring degradation. It has been recently demonstrated that CM and, especially, BC have a remarkable capacity to sorb metribuzin, allowing to speculate that the addition of composted and carbonaceous materials to the soil may greatly enhance the adsorption of this compound (Loffredo et al. 2019).

The presence of plants did not modify the trend of metribuzin along the soil profile but significantly reduced its quantities at various depths of all treatments, especially in the upper 5 cm of soil where most of the roots were present (Fig. 4b). On average, for the treatments, in 25 days, hemp removed a significant amount of metribuzin from the whole column, resulting more than twice that disappeared in bare soil (Table 3). This can be reasonably attributed to both plant uptake and enhanced microbiological degradation. The presence of CM and BC did not affect significantly metribuzin disappearance neither in bare soil nor in soil with hemp (Table 3). This is in contrast with what reported by Mehdizadeh et al. (2019), who found that a green CM could promote the decay of this herbicide in soil, mostly for the stimulation of degrading microbes. Benoit et al. (2007) studied the pathways of metribuzin degradation in soil and concluded that biodegradation is the foremost process.

Residual BPA was uniformly distributed along the first 15 cm of soil only, whereas it accumulated mainly in the upper layers (0–10 cm) in soil + BC (Fig. 5a). Also for this compound, the retention and transport in soil is strictly affected by the level of soil organic matter (Shi et al. 2019). Hemp plants did not modify the vertical distribution of BPA in soil but were able to significantly reduce residual BPA in the upper 10 cm of the soil, compared with bare soil, contrasting leaching (Fig. 5a). Maximum residual BPA was found in the upper 5 cm of soil + BC and soil + BC + plants, where BPA concentrations were, respectively, 5.37 and 4.75 μg g^−1^. Statistical analysis of the data of BPA disappearance in soil excluded the importance of the addition of CM or BC both in bare and in planted soil, whereas plants did play a relevant role in the process (Table 3). Our results are in agreement with what reported by Xu et al. (2015) who found that BC noticeably reduced BPA mobility and leaching in soil but did not affect its degradation.

Both E2 and OP were the least mobile in the soil, with little changes between soil only and soil + CM. (Fig. 5b, c). A high retention of the two EDCs in soil, and especially the contribution of BC, was expected on the basis of the log *K*_ow_ of E2 (3.90) and OP (5.50). At the end of experiments, residual E2 in the 10–15 cm layer of soil only and soil + CM was lower or much lower than that found for BPA and the two pesticides (Fig. 5b). Tong et al. (2019) showed that adsorption of E2 in soil was highly depended on mineral-organic complexes of soil. Hemp did not alter substantially the distribution of E2 along the soil profile; however, it significantly reduced its residue in the upper 0–5 cm layer of amended soil (Fig. 5b). On average, for the treatments, the disappearance of E2, after 25 days, in the whole soil column, although not affected by the amendment, was much greater in planted soil (21.4%) than in bare soil (12.8%) (Table 3), indicating a marked contribution of hemp in the loss of this compound. It is reasonable that both plant uptake and enhanced microbiological degradation played a role in E2 disappearance.

Almost all residual OP accumulated in the upper 10 cm of soil only and soil + CM or even in the upper 5 cm of soil + BC, evidencing the very low potential of OP to leach (Fig. 5c). Compared with bare soil, planted soil showed a similar vertical distribution of OP, reducing significantly the residual compound only in the upper 5 cm of the unamended soil but excluding OP presence below 10 cm in the treatment soil + BC (Fig. 5c). At the end of experiments, the lowest quantity of OP in the whole soil column was measured in the treatment soil + plants (Table 3). Although neither CM nor BC affected OP disappearance in bare soil, both materials significantly decreased OP removal by hemp (Table 3). Possible explanations for this could be both the strong retention of OP by the materials that reduced OP bioavailability and competed with the possible root uptake and the lesser availability of root exudates for the rhizodegradation process. In fact, even some plant metabolites, such as phenolic acids, might have been adsorbed by the materials and be therefore less available for soil microorganisms. Zhou (2006) evidenced the importance of organic colloids in controlling the environmental fate of OP. The strong sorption of OP on a red spruce BC was demonstrated (Loffredo and Taskin 2017).

Results obtained in these soil column experiments evidenced the crucial role of the upper 5-cm layer of soil, where most of the rhizosphere is located and most of the aerobic processes occur. Therefore, we tried to relate the residual compounds in this layer, at the end of the experiments. with important properties of the molecules. Plotting residual compounds in the upper 5 cm of soil versus the corresponding log *K*_ow_, significant positive correlations (*P* ≤ 0.05) were found only for unamended soil, both bare and planted. This indicated that the leaching of the five compounds in soil was inversely related to their hydrophobicity and that the addition of CM or BC could alter this law, possibly because it acted simultaneously on more processes, like retention, movement, plant uptake, and degradation, which are not strictly related to hydrophobicity. Correlations between the residual compound in the upper 5 cm of soil, soil + CM and soil + BC (bare soil), and the corresponding organic matter (OM) content were significant (*P* ≤ 0.05) for metribuzin, BPA and E2. In this case, let us assume that, for the latter compounds, retention was the prominent process to contrast leaching or that both leaching and degradation were influenced with similar extent and trend. Differently, in the cases of the very soluble metalaxyl-M and the highly hydrophobic OP, the OM content might have influenced differently leaching and biodegradation or it was not the foremost property.

Finally, the plant removal of each compound from the whole column, calculated as the difference between the amount which disappeared on average in planted soil and bare soil (Table 3), and excluding OP, was inversely related to the hydrophobicity of the molecule, being the least hydrophobic the most removed. In the case of OP, it is possible that its overall surface localization, which coincided with the zone of the greatest root expansion, favored plant removal beyond what was expected. Based on the hydrophobicity of OP and the absence of OP residues in plant tissues (see the “Plant response and compound accumulation” section), it is plausible that OP removal occurred mainly or exclusively through rhizodegradation.

#### Plant response and compound accumulation

**Fig. 6:**
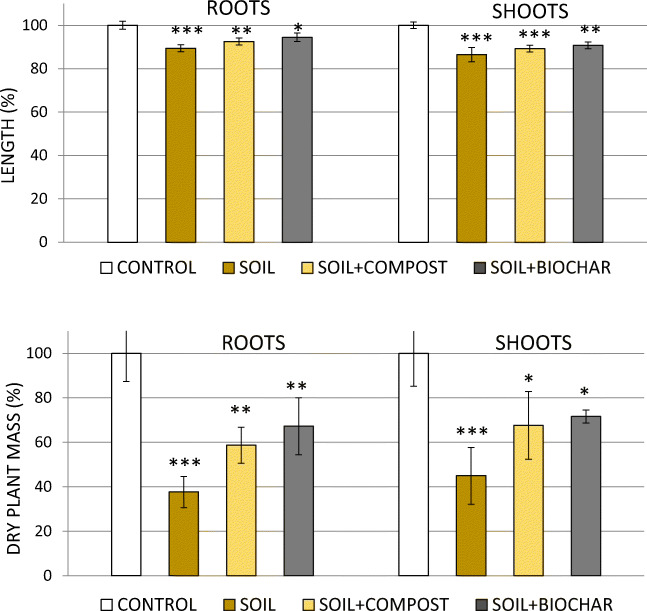
Effects of the substrate on the elongation and dry mass of hemp plants grown in soil columns compared with controls (plants grown in not contaminated soil). Means were separated by the least significant difference (LSD) test. *P ≤ 0.05, **P ≤ 0.01, ***P ≤ 0.001. The vertical line on each bar indicates the standard error (*n* = 3)

Hemp plants grown for 25 days in multi-contaminated soil not amended or amended with CM or BC did not show visual alterations, except a delayed growth, compared with plants grown in uncontaminated soil (control). Biometric data showed lower root and shoot elongation and fresh and dry biomass, denoting an unquestionable toxicity of the chemical mixture on plants (Fig. 6). Phytotoxicity of these compounds, individually applied, was already observed in previous studies. Teixeira et al. (2011) reported root depression and other damages on plant *S. nigrum* L. exposed at concentrations ≥ 12.5 μg g^−1^ of the sole metalaxyl in 28 days. Doses of 4.6 and 46 μg g^−1^ of BPA altered the root morphology and markedly reduced fresh weight of 16-day seedlings of ryegrass and radish (Loffredo et al. 2010).

However, contaminant toxicity on hemp was significantly attenuated by the presence of CM and, especially, BC which, in the order, increased the dry weight of roots by 55 and 78% and of shoots by 51 and 59%, compared with soil only (Fig. 6). These results clearly indicated the occurrence of plant-protective effects by both materials.

**Fig. 7:**
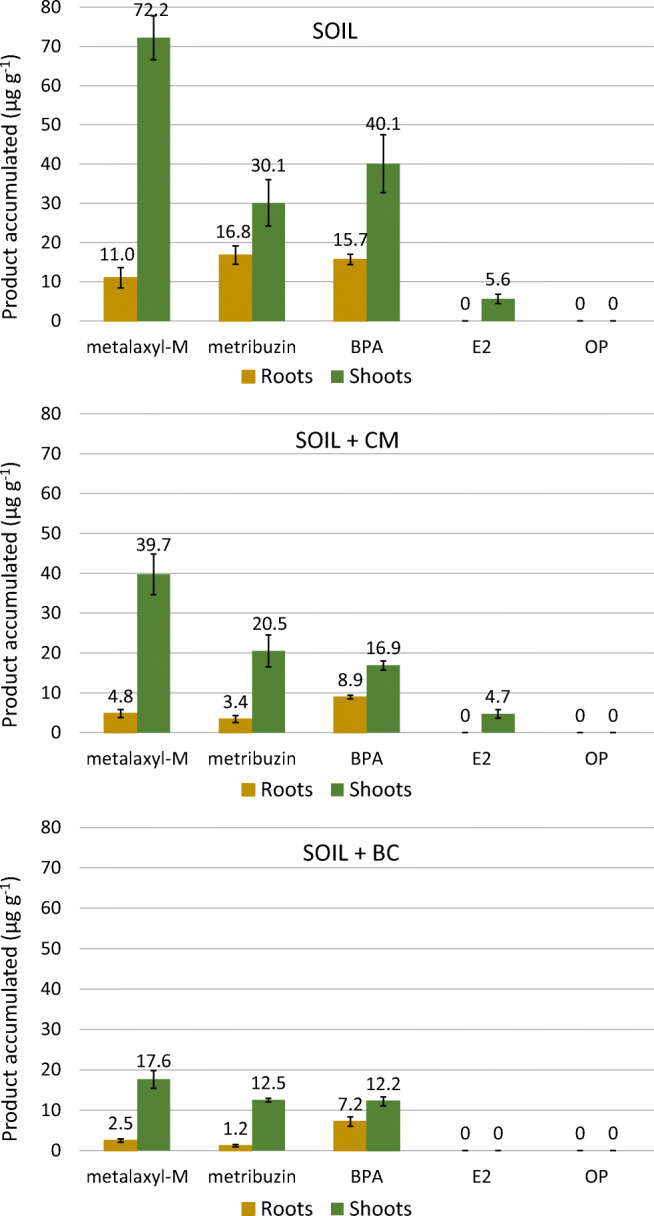
Compound accumulation in root and shoot dry mass of hemp grown for 25 days in columns filled with soil only and soil added with CM or BC. The vertical line on each bar indicates the standard error (*n* = 3)

As already observed in germination experiments, hemp showed a great ability to absorb metalaxyl-M from the soil and accumulate it, especially in the aerial part (Fig. 7). The maximum compound accumulation was measured in the shoots of the plants grown in unamended soil, being 72 μg g^−1^ (Fig. 7). Similar to what reported by Teixeira et al. (2011), a much lower accumulation of metalaxyl-M occurred in hemp roots. The preferential translocation of this compound from roots to shoots can depend on the low hydrophobicity of this compound that allows high mobility into the vascular system of plants. Kubicki et al. (2019), studying the dynamic of metalaxyl in tomato, reported that the compound was readily taken up by the roots with the normal water absorption and translocated uniformly to the aerial organs through the xylematic vessels. Very recently, Gong et al. (2020) found that metalaxyl-M translocated rapidly in chrysanthemum plants where it accumulated mainly in the leaves and ascribed that to the relatively low log *K*_ow_.

In soil + CM and soil + BC treatments, plants accumulated less metalaxyl-M and always more in the shoots than in the roots (Fig. 7). These results confirmed the evidence of a straight relationship between the quantity of compound removed and that of compound accumulated. Anyway, if on the one hand CM or BC hindered the removal of metalaxyl-M by plants, on the other hand, they clearly exerted an antitoxic activity that was crucial for allowing plants to tolerate heavily polluted soil. The percentage of metalaxyl-M accumulated in plants, compared with that removed from the soil, was very low and minimum in soil + BC treatment (Table 4). This suggested that BC, in addition to influencing absorption and accumulation, could also affect the plant transformation of this molecule. Further studies could elucidate this aspect.

**Table 4:** Percentage of compound accumulated in plant mass compared with the quantity removed by plants from the soil

Compound	Soil	Soil + CM	Soil + BC
Metalaxyl-M	2.34 ± 0.12 b	3.14 ± 0.21 a	1.04 ± 0.10 c
Metribuzin	1.55 ± 0.10 a	1.22 ± 0.18 ab	1.13 ± 0.03 b
BPA	2.52 ± 0.09 a	1.66 ± 0.09 b	1.32 ± 0.09 c
E2	0.41 ± 0.12	0.41 ± 0.12	n.d.
OP	n.d.	n.d.	n.d.

Data were analyzed by ANOVA and means were separated by LSD test at *P* ≤ 0.05 (*n* = 3)

*n.d.* not detected

The herbicide metribuzin accumulated in plants to a lesser extent than metalaxyl-M and once more especially in shoots (Fig. 7). Moreover, the extent of accumulation depended on the soil treatment. Preferential accumulation of metribuzin into the aerial plant organs might depend on the relatively low hydrophobicity of this compound and the high mobility into the plant vascular system. The lowest amounts of metribuzin (12.5 μg g^−1^) were found in the roots and shoots of plants grown in the presence of BC and that might be ascribed to the lower removal by plants of this treatments (Fig. 7). The percentage of metribuzin accumulated in plants compared with that removed was generally very low and significantly lower in soil + BC, compared with soil only (Table 4). Also for this compound, it seemed that BC somehow stimulated the transformation of metribuzin into the plant tissues.

Evidence of the capacity of hemp to absorb BPA from soil was confirmed by the significant product accumulation found in the shoots and roots of hemp (Fig. 7). A lower amount of BPA was extracted from hemp roots than from shoots (Fig. 7). Compared with metalaxyl and metribuzin, the ratio between the compound accumulated in roots and that accumulated in shoots was higher for BPA. That might depend on the higher hydrophobicity of this molecule that made translocation more difficult. Also for BPA, plant metabolization seemed to be influenced by the addition of the materials, in which accumulation is lower in the presence of CM and, especially, BC (Table 4).

The limited amount of E2 found only in the plant shoots of unamended soil and soil + CM (Fig. 7) can be ascribed to the preferential transport of this molecule to the upper organs. Chuang et al. (2019) demonstrated that E2 was rapidly and conspicuously absorbed by lettuce but its accumulation in roots was negligible because the compound was preferentially transported upwards to shoots with the water flow. The same authors reported a very rapid metabolization of E2 by plants, mainly in the green organs, with the mass recovery of less than 25% after only 48 h of exposure (Chuang et al. 2019). Anyway, considering the quantity of E2 removed on average by plants (Table 2) and the very low percentage of contaminant accumulated in plant tissues (Table 4), we can hypothesize that another process might have contributed to these results, namely rhizodegradation. Root exudates released by hemp, and localized especially in the upper layer where E2 was more concentrated, might have stimulated the activity of microorganisms involved in E2 degradation. Furthermore, the presence of BC in the soil might have promoted rhizodegradation and that could be the reason why E2 was not extracted from plants grown in soil + BC. A recent study demonstrated that *Bjerkandera adusta*, a common soil-resident fungus, has a relevant capacity to degrade E2 and that this activity is even higher in the presence of BC (Loffredo et al. 2016).

The OP was the only molecule that was not found in the plants, either in roots or in shoots, of all treatments. The hydrophobic character of this molecule must have played an important role in reducing the mobility of this compound both in soil and in plants. Comparing residual OP in bare and planted soil (Table 3), it was evident that an appreciable disappearance of OP could be ascribed to hemp. To explain these results, we can appeal to at least two reasons. The first is that hemp absorbed OP and efficiently transformed it. In fact, although the high log *K*_ow_ (5.50) of OP seems to hinder plant absorption, it was demonstrated that plants like *Portulaca oleracea* can efficiently absorb and metabolize OP and other EDCs, including BPA and E2 (Imai et al. 2007). The second is rhizodegradation. Similar to what possibly happened for E2, the presence of plants and their root exudates might have stimulated microbial degradation of OP in the soil. That might have been even more important for OP than E2, considering the very superficial localization of OP in the soil. Phenolic EDCs, such as OP, can be effectively degraded by soil-resident ligninolytic fungi (Loffredo et al. 2016).

## Conclusions

Seedlings of *Cannabis sativa* L. proved to be very effective in removing individually the fungicide metalaxyl-M and the endocrine disruptor BPA from water. Despite that both molecules showed toxicity on seedlings, their removal was relevant up to a concentration of 50 μg mL^−1^ of metalaxyl-M and 100 μg mL^−1^ of BPA. Residual compounds accumulated in hemp tissues in 7 days were much lower than the amounts removed from the medium, indicating an efficient metabolization of these molecules. When hemp was allowed to germinate and grow in columns filled with a soil multi-contaminated with metalaxyl-M, metribuzin, BPA, E2, and OP, despite the toxic effects, the plant once more showed a noticeable remediation capacity. The addition to the soil of materials like CM and BC relieved, at least partly, the phytotoxicity stress, albeit the plants removed less quantities of metalaxyl-M and OP. Both CM and, especially, BC were able to modify the leaching pattern of the compounds along the soil profile, favoring their localization in the upper layers, compared with unamended soil. Among the five compounds, only OP was not found in the plants, whereas the others, especially the less hydrophobic pesticides, were absorbed by hemp and accumulated mainly in the aboveground organs. A very large part of the compounds taken up by plants was transformed in plant tissues. The overall findings of this study suggest that hemp is a promising candidate for practical phytoremediation of wastewater and soil from pesticides and endocrine-disrupting chemicals.
